# 

**Published:** 1890-05

**Authors:** 


### EDITORIAL.

### Seventh District Dental Society—Ohio.

The second annual meeting of this society will be held in Hamilton, Butler County, on Tuesday, the 27th of May, at 10 o'clock A. M., continuing through the day and evening.

The territory of this society embraces about a dozen counties in the southwest corner of the State. It is auxiliary to the State Society, and it is to be hoped that all members of the profession in this district will become interested in the society, attend its meetings, and take an active part in its work.

There will be a number of papers read and discussed, new and valuable appliances exhibited; there are in addition matters of general interest that ought to receive attention at the hands of these district societies.

The regular time for the meeting is the third Tuesday of May, but from the fact that several of the members will be absent on that day the Executive Committee thought it best to defer this meeting one week. Let all dentists in this district, who have any interest in, or desire for the welfare and progress of their chosen profession, be present at that time. A programme will soon be issued.

### Iowa State Dental Association.

The twenty-eighth annual meeting of this society will be held in Dubuque, Iowa, May 6, 7, 8 and 9. This is one of our energetic, efficient State societies, one that has done very much for the profession in Iowa. In the announcement which has just been issued it is stated that no labor or expense has been spared to make this meeting of inestimable value to every dentist. A programme of unusual interest has been provided and all who are interested in progressive dentistry will find a feast of knowledge awaiting them. The programme has been arranged on the basis of practicability; ample provision has been made for clinics and demonstrations.

The character of the meetings of this society has been such as to enlist the interest and presence of many prominent dentists from outside the State and the meeting of this year will doubtless not be an exception in this respect, so that a large, interesting and instructive meeting may confidently be anticipated.

### American Medical Association.

The annual meeting of the American Medical Association will take place at Nashville, Tenn., beginning May 20th, and continue four days. This will undoubtedly be a very large meeting of this body, as it is at a central point so far as the whole country is concerned. It is to be hoped that a large number will be present, as has been expressed by another, to carry with them a cordial, social greeting, sandwiched with scientific papers and bubbling over with observations to be let loose in discussion.

Many men do not go to these annual meetings because they think they can't spare the time or necessary expenses. Very often the apology is a mere excuse. The men who go are nearly always gainers; their hearts are enlarged, their vision expanded, their intellects are brightened and fed, while their whole being is freshened. The time is not lost but well spent, and the financial investment uniformly returns a full per cent.

As most of the readers of the REGISTER know, there is in connection with this body a dental section, for which by reference to the April number of the REGISTER it will be seen a good programme has been prepared, and we sincerely hope there will be a large representation of dentists at that meeting. They have not only the advantage of the work done in the section, but of that done in the whole body, and that done in other sections so far as their time and inclination may permit. The opportunity to meet and associate with the leading minds of the medical profession in this country is a matter not to be lightly esteemed, and we hope that so far as possible, dentists will avail themselves of this opportunity.

### A New Medical Dictionary.

There has just been issued from the publishing house of P. Blakiston, Son & Co., of Philadelphia, a new medical dictionary, by George M. Gould, Ophthalmic Surgeon of the Philadelphia Hospital, Clinical Chief Ophthalmological Department, German Hospital, Philadelphia.

### Northern Ohio Dental Association.

The thirty-first annual meeting of this body will be held in Canton, Ohio, Tuesday, May 13th, 1890, at 10 o'clock A. M. and continue three days. A cordial invitation is extended to all the profession. An interesting and attractive programme of subjects has been prepared for that occasion, and a number of papers on practical subjects will be read and discussed. The first paper on Combination Fillings, will be by Dr. Charles R. Butler; the second, on Dentistry of To-Day, by Dr. George H. Wilson; the third, on Causes of Recurrence of Decay of the Teeth, by Dr. E. J. Way.

Arrangements have been made for probably two sessions of clinics. This will be an occasion that certainly ought to call out the entire profession of northern Ohio.

---

TREATMENT OF A COMMON "COLD."—Salicylate of sodium in free doses, gives as satisfactory results in the treatment of "bad colds" as it does in cutting short tonsillitis. Sodii salicylatis, ℥ss; syr. auranti cort., ℥ss; aquæ menth. piper, ad ℥iv. M. Sig. A dessert-spoonful every three or four hours. A dose every three hours until a free specific influence of the salicylate—tinnitus aurium—is observed—will so far control the symptoms that the aching of the brow, eyes, nose, etc., will cease. The sneezing and "running from the nose" will also abate and will disappear in a few days, not leaving, as is usual under other treatments, a cough, from the extension of the inflammation to the bronchial tubes.

Apropos: Dr. Philpots writes the *British Medical Journal* that when sneezing manifests itself, before any inflammatory action has been established, a single local application of salicylic acid to the irritated lining membrane of the nose will be quite sufficient, in the majority of cases, to abort a common cold. The formula suggested is: Sodii salicylatis ʒiv; acid. boracis (pulv.) ℥j; cocainæ hydrochlor. gr. xxij. M. Mix by agitation; not in a mortar. Snuff or draw into the nose, or preferably, use with insulator.—*Memphis Med. Journal.*

### CONTENTS.

COMMUNICATIONS.

THE DENTAL REGISTER,

A MONTHLY MAGAZINE DEVOTED TO THE INTERESTS OF THE

**DENTAL PROFESSION.**

*Terms Reduced to* $2.00 *Per Year. Single Copies,* 25 *Cents.*

**EDITED BY PROF. J. TAFT, D.D.S.**

—AND—

**Published by SAM'L A. CROCKER & CO., Ohio Dental and Surgical Depot.**

The volume commences in January and closes in December.

The REGISTER being issued monthly is always fresh, therefore Indispensable to the practitioner who desires to keep posted up with the new inventions and improvements which are daily developed. Neither expense nor effort will be spared to give value and variety to its contents. Articles will be illustrated with well executed engravings whenever they will add to the interest or assist to explanation.

Its extensive circulation and frequency of issue make it one of the BEST DENTAL ADVERTISING MEDIUMS in the United States.

All subscriptions must begin with January or July.

Local or special notices, five lines or less, will be inserted for $3 each insertion; over five lines, 50 cts. per line.

All advertising bills payable in advance, or at furthest, after the issue of the first number containing the advertisement. All transient advertisements to be paid for in advance.

☞CONTRIBUTIONS SOLICITED.

Exclusively Editorial Communications should be addressed to the Editor.

The latest number of the REGISTER may be ordered with or without other goods at any time, and all letters should be addressed to

**SAM'L A. CROCKER & CO., Ohio Dental and Surgical Depot, Cincinnati, O.**

